# Supplementary material for: Complex‐centric proteome profiling by SEC‐SWATH‐MS
Source: Mol Syst Biol. 2019 Jan 14;15(1):e8438. doi: 10.15252/msb.20188438 (PMC6346213; doi:10.15252/msb.20188438)
Supplement: Supplementary file 6 — Dataset EV5 [file MSB-15-e8438-s006.zip › feature_plots_corum/632-2.pdf]

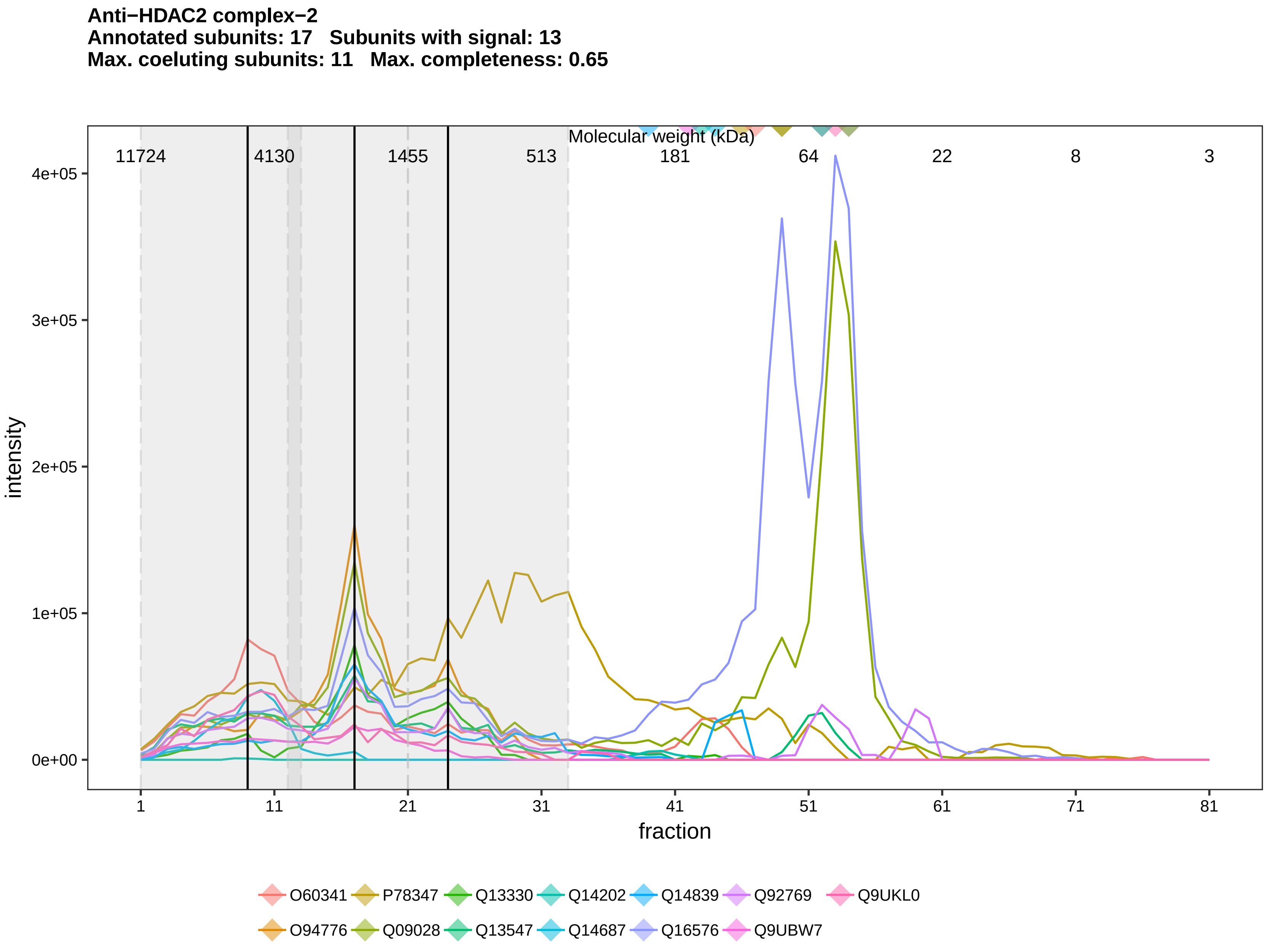
Anti−HDAC2 complex−2
Annotated subunits: 17 Subunits with signal: 13
Max. coeluting subunits: 11 Max. completeness: 0.65
Molecular weight (kDa)
11724
4130
1455
513
181
64
22
8
3
intensity
4e+05
3e+05
2e+05
1e+05
0e+00
fraction
1
11
21
31
41
51
61
71
81
O60341
P78347
Q13330
Q14202
Q14839
Q92769
Q9UKL0
O94776
Q09028
Q13547
Q14687
Q16576
Q9UBW7
